# Observations on the Use of the Lichen Islandicus, or Iceland Liverwort, in Consumptions of the Lungs

**Published:** 1807-03

**Authors:** James Lyons

**Affiliations:** Richmond, America


### Observations *on the* Use *of the* Lichen Islandicus, *or* Iceland Liverwort, *in* Consumptions *of the* Lungs, *By* James Lyons, *M. D. of Richmond, America.*

In the successful treatment of a disorder so common and so fatal to the most promising part of the human species, viz. the youth of both Sexes, as the phthisis pulmonalis, or consumption, I consider every remedy which has appeared from experience to promote a cure, as worthy public notice, and I shall not therefore offer any apology for the subsequent communication. In the spring of last year, 1805, Miss C. D. about 20 years of age, of a constitution remarkably delicate, tall and thin in her person, with a fair florid complexion, after having been troubled most of the preceding winter with an almost constant cough, was confined by a violent pain in her side, frequent chills, and continued fever, while her cough became incessant, unless checked by anodynes, assisted by warm, softening, diluting drinks. I bled her twice, and took away each time as much as her weak condition permitted. She had repeated blisters applied to her sides and breast, and used every other auxiliary remedy suggested by Dr. M'Clurg, who visited her, or by her friends. She, however, continued to decline till her recovery was despaired of while she remained here, and a change of climate, and a voyage to sea, were thought the only resources. These, from circumstances, could not, at that time, be accomplished; she became so reduced as to be confined constantly to her bed, had frequently chills and fevers, pains in both sides so acute, that she could not lie on them, and her death was speedily expected. At this period it was recollected, that Mr. Carnillon, a French gentleman, who left this place in 1804, had sent to Mr. Cherallic, as a farewell present, a box of the Lichen Islandicus, or dry Iceland moss, with strong commendations of the use of it in pulmonary complaints. It was prepared according to the following directions, which accompany it:—" Take an ounce and an half of the dry Iceland moss, wash it in hot water, boil it in a quart of hot water till reduced to a third, and strain it. Of the decoction give three or four glasses, suppose wine-glasses, in the course of the day, at convenient intervals. If any advantage can be derived from the use of milk, and the stomach can bear it, instead of water, make the decoction with an equal quantity of milk and water, in the same manner, and add, at pleasure, sugar, or the syrup of lichen."

I had a few days before commenced the trial of the tincture of digitalis, or fox-glove, with her, which I thought had been of service in some previous cases; but this tincture disordered her head so much, that it was necessary to omit it, or to give it in very small doses, as ten or twelve drops once or twice a day, as she could bear it. Under this treatment she soon began to get better, and, in the course of a month, was able to ride in a carriage; after which she rapidly recovered so completely, as to gain her health more firmly than it was before the attack. I have since used the lichen-milk with Mr. J. W. whose situation was similar to that of Miss D. by the assistance of which he was, in a short time, enabled to leave this place for

Norfolk,

Norfolk, the air of which was thought preferable for him. The benefit from the Lichen Islandicus in the last case, confirmed my opinion of its efficacy in the first. It makes, when boiled sufficiently, a mucilaginous bitter, not unlike our horehound, into which something like gum-arabic is dissolved. The Lichen Islandicus is a remedy unknown to me till the last year, and it is probably not familiar to most other medical practitioners in America. From the experience I had of its good effects, I think it is well worthy attention, and solicit those physicians who may have occasion, and an opportunity of using it, to give it a trial, and communicate the result to the public.

*Virginia, Feb.* 13, 1806.

